# Spatial distribution of Intra-retinal Hyper-reflective foci and impact on progression in eyes with intermediate Age-related macular degeneration

**DOI:** 10.1007/s00417-025-06964-w

**Published:** 2025-09-25

**Authors:** Aditya Verma, Muneeswar G. Nittala, Jonathan L. Haines, Margaret A. Pericak-Vance, Dwight Stambolian, SriniVas R. Sadda

**Affiliations:** 1 Doheny Eye Institute, Pasadena, CA USA; 2 Department of Ophthalmology and Visual Sciences, University of Louisville, Louisville, KY USA; 3 Cleveland Institute for Computational Biology, Department of Population & Quantitative Health Sciences, School of Medicine, Case Western Reserve University, Cleveland, OH 44106 USA; 4 John P. Hussman Institute for Human Genomics, University of Miami Miller School of Medicine, Miami, FL, Florida 33136 USA; 5 Department of Ophthalmology, Perelman School of Medicine, University of Pennsylvania, Philadelphia, PA 19104 USA; 6 Department of Ophthalmology, David Geffen School of Medicine, University of California, Los Angeles, Los Angeles, CA USA; 7 David Geffen School of Medicine, Doheny Eye Institute, University of California Los Angeles, 150 N, Orange Grove Blvd, Pasadena, CA 91103 USA

**Keywords:** Age-related macular degeneration, Intraretinal hyper-reflective foci, Optical coherence tomography

## Abstract

**Aim:**

To analyze the longitudinal spatial distribution characteristics of intraretinal hyperreflective foci (IHRF) in eyes with intermediate age-related macular degeneration (iAMD).

**Methods:**

The optical coherence tomography (OCT) data was collected from the Amish Eye Study, for all patients with evidence of iAMD and IHRF at baseline, who completed a 24 month (M24) follow up visit. Early Treatment Diabetic Retinopathy Study (ETDRS) rings were placed on the enface scans from all volume scans showing IHRF, and the frequency and the density of IHRF was measured in the central subfield (CSF; 1 mm circle), the 1–3 mm ring (parafoveal region) and the 3–6 mm ring (perifoveal region). The distance of all IHRF lesions from the foveal center was measured.

**Results:**

Forty-nine eyes (40 subjects) had iAMD and IHRF at baseline and M24. 23 eyes (46.9%) showed progression to late AMD. The frequency of IHRF was greatest in the parafoveal ring, although the density was highest in the CSF. IHRF number and density numerically increased in all regions at M24, though the increase was significant only in the perifoveal region. Although regression analysis did not show a relationship between baseline IHRF distribution and risk for progression to late AMD at 2 years, eyes that developed late AMD at M24 tended to show a more eccentric distribution of IHRF over time.

**Conclusions:**

IHRF do not distribute evenly across the macula in eyes with iAMD, with a higher density centrally, but greater frequency parafoveally. IHRF tend to distribute more peripherally over time, however, among eyes that progress to late AMD. These observations may provide new insights into the pathophysiology of these lesions.

## Key messages

*****What is known before***:**

- Intra-retinal hyper-reflective foci (IHRF) are an Optical Coherence Tomography (OCT)-based biomarkers known to increase the risk of progression of intermediate AMD (iAMD) to advanced stages, especially those in close proximity to the retinal pigment epithelium (RPE).
- The axial distribution pattern shows that these lesions increase in frequency over time, predominantly accumulating in the outer retinal layers.

*****What this study adds***:**

- The transverse spatial distribution of IHRF in the macula of eyes with iAMD shows the highest density in the central 1mm region, though over time, the density increases more eccentrically.
- Although the baseline distribution of IHRF does not impact the risk for AMD progression, eyes that progress to late AMD tend to have a more eccentric distribution of IHRF over time.

## Introduction

Intra-retinal hyper-reflective foci (IHRF) are established optical coherence tomography (OCT)-based risk factors in the cascade of events leading to progression of age-related macular degeneration (AMD) from early to advanced stages [1–13]. In the context of AMD, they are generally hypothesized to originate from the degenerating retinal pigment epithelium (RPE), although an inflammatory origin, particularly in the context of neovascular disease, has also been conceptualized by some investigators [9, 14–18]. It is important to note that HRF in the context of dry AMD are distinct from HRF in the context of diabetic macular edema, where they typically correlate with lipid exudates or inflammatory/glial cells. To further validate the concept of RPE origin, various RPE histological phenotypes have been correlated with IHRF lesions based on the reflectivity patterns on OCT-B scans [19–21].

More pertinent is the concept of inner retinal migration of these pigment lesions, which is known to be intricately associated with progression to RPE and photoreceptor atrophy in these eyes [9, 16, 22–25]. Clinicopathological correlation studies have suggested the origin of such migrating pigment cells from the underlying degenerating RPE layer [19, 20]. Particularly in eyes with intermediate AMD (iAMD, i.e., eyes with large drusen or pigmentary abnormalities) [26], the frequency and density of these lesions increases over time, migrating inwards from the outer nuclear layer [22, 27]. Previous studies indicate that once formed, these IHRF lesions may indicate a critically distressed RPE, leading to clustering of these lesions in close proximity to the RPE, and abetting an increased risk of progression to late AMD (eyes with neovascular component or geographic atrophy) over time [12, 16, 22].

Although studies have investigated the axial distribution pattern of IHRF and their impact on progression to advanced stages over time, few studies have explored the transverse or enface distribution of such lesions across the macula. A post-hoc analysis of fellow iAMD eyes from the HARBOR trial reported that the greatest mean thickness of IHRF was located 0.5 mm temporal to fovea, and this significantly increased the risk of progression to macular atrophy. Beyond 1.5 mm eccentricity, the mean thickness of HRF decreased, and the risk of progression to atrophy was no longer observed [8]. A recent study by Saßmannshausen et al. similarly confirmed the predominance of these lesions at around 0.5 to 1.5 mm from the foveal center [28].

While these studies revealed a very interesting and homogenous distribution pattern of IHRF in the macular region, several pertinent questions remained unanswered. How does this distribution pattern change over time? Does this have any impact on the progression of these eyes to late AMD? To answer these residual questions, our study aimed to evaluate the transverse distribution pattern of IHRF in the macular region in a cohort of eyes with iAMD from the Amish Eye Study (AES), and to determine if the distribution impacted the risk of progression to advanced stage AMD over a 24-month period [7, 11, 12, 16].

## Methods

### Study overview

The data was collected from the ongoing AES (supported by NEI R01EY023164 and 1R01EY030614), a prospective longitudinal observational study aimed at evaluating OCT-based risk factors and their genetic association with AMD progression [7]. This institutional review board (IRB) - approved study was conducted in accordance with the Health Insurance Portability and Accountability Act and adhered to the tenets of the Declaration of Helsinki. All subjects signed written informed consent. Eligible subjects had evidence of iAMD based on Beckman classification [26] and IHRF present on the baseline OCT scans. Late AMD was determined by the presence of macular neovascularization or geographic atrophy evident on clinical exam and/or imaging at M24. Eyes with gross segmentation errors which could impact retinal slab selection, poor-quality images, and diseases other than iAMD were excluded from this analysis. OCT volume scans of both eyes of all subjects were acquired using the Cirrus OCT (Carl Zeiss Meditec, Dublin, CA; 512 × 128 macular cube; 6 × 6 mm scan region centered at the fovea). Deidentified OCT volumes were analyzed at the Doheny Image Reading and Research Lab (DIRRL) by trained graders.

### OCT Analysis Protocol (Fig. 1)

**Fig. 1:**
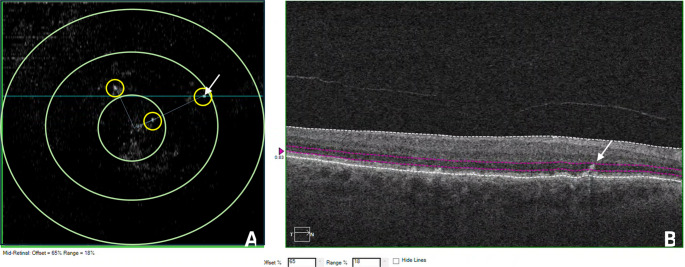
OCT analysis protocol: ETDRS grid circles (1 mm, 3 mm and 6 mm diameter) were placed on the enface OCT scan. In the present example, only the outermost of the sequential enface slabs used to assess IHRF is shown. Frequency of IHRF was assessed in the various regions within the ETDRS grid. Foveal center was marked and the distance of each IHRF was measured using the caliper tool. The same process was repeated in each B scan demonstrating an IHRF lesion and isolating the overlying lesions, which was confirmed on the adjacent structural B scan

**Fig. 2:**
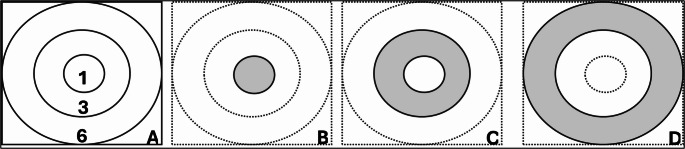
Early Treatment of Diabetic Retinopathy grid (ETDRS) regions (shaded gray) superimposed on a 6 × 6 mm macular area (Fig A). Fig B represents the central 1 mm region (central subfield, CSF); Fig B represents the 1–3 mm ring (parafoveal ring); and Fig D represents the outer 3–6 mm ring (perifoveal ring)

Using a previously described approach [12, 16], all the IHRF lesions were identified on multiple sequential enface slabs of the mid-retina generated from the macular cube (128 B-scans, each containing 512 A-scans) as previously described. Multiple sequential slabs are required because in some cases, multiple IHRF may be present at different axial depths along a single A-scan, and the use of a single slab would result in undercounting IHRF lesions. The innermost boundary of the sequential slabs is located at the outer border of the nerve fiber layer and the outer boundary is located immediately above the inner surface of the RPE. The IHRF lesions were defined as well-circumscribed hyper-reflective lesions within the neurosensory retina at least 3 pixels in size. The foveal center was identified by navigating through the structural OCT B scans of the volume, and the same position was identified on the corresponding enface image. The distance of each HRF lesion to the foveal center was then measured using the caliper tool available in the Cirrus software for each enface slab, wherever the IHRF were identified. Early Treatment Diabetic Retinopathy Study (ETDRS) rings were drawn using the “add circle” option, which adds a 1 mm diameter circle, centered at the image center (central subfield, CSF). Once this circle was confirmed to be centered at the fovea center, 3 mm and 6 mm diameter rings were subsequently added also centered at the fovea. Each enface slab was carefully inspected for the IHRF lesions, which were confirmed on the corresponding structural B scan, and counted. The frequency of all IHRF lesions in the CSF (1 mm circle), 3 mm ring (outside the CSF, parafoveal), and 6 mm ring (region outside the 3 mm ring, perifoveal) was determined [figure 2]. In addition, as these regions are not equal in size, the density of IHRF in each region was also calculated by dividing the frequency by the area of that region (i.e., 0.785 mm^2^ for CSF, 6.28 mm^2^ for parafoveal ring, and 21.195 mm^2^ for perifoveal ring).

### Statistical analysis

All relevant analyses were performed using statistics software (SPSS Statistics v 21.0; IBM Corp., New York, NY, USA). The frequency and the density in each ETDRS region and the distance from the foveal center at baseline and at 24-months were analyzed and compared using paired t-tests. Descriptive statistics were reported as mean ± standard deviations, wherever applicable. The normalcy of distribution of data was confirmed using the Shapiro-Wilk test. Univariate logistic regression was used to analyze the relationship between the distance of IHRF from the foveal center at baseline, and the development of late AMD at M24. The value of *P* < 0.05 was considered statistically significant.

## Results

At baseline, 1339 subjects underwent volume OCT assessment, and 666 subjects returned for a M24 follow-up visit. One hundred and twenty eyes of 71 subjects had evidence of iAMD at baseline. Of these, 49 eyes (40.8%) from 40 subjects showed evidence of both iAMD and IHRF, at both baseline and at the 24-month follow-up. This group constituted the final analysis cohort for this study [mean age ± SD; 74.17 ± 9.37 years; 24 (60%) females]. Of these 49 eyes, 23 eyes (46.9%) showed progression to late-stage AMD (20 eyes developed geographic atrophy, whereas 3 eyes developed macula neovascularization).

**Fig. 3:**
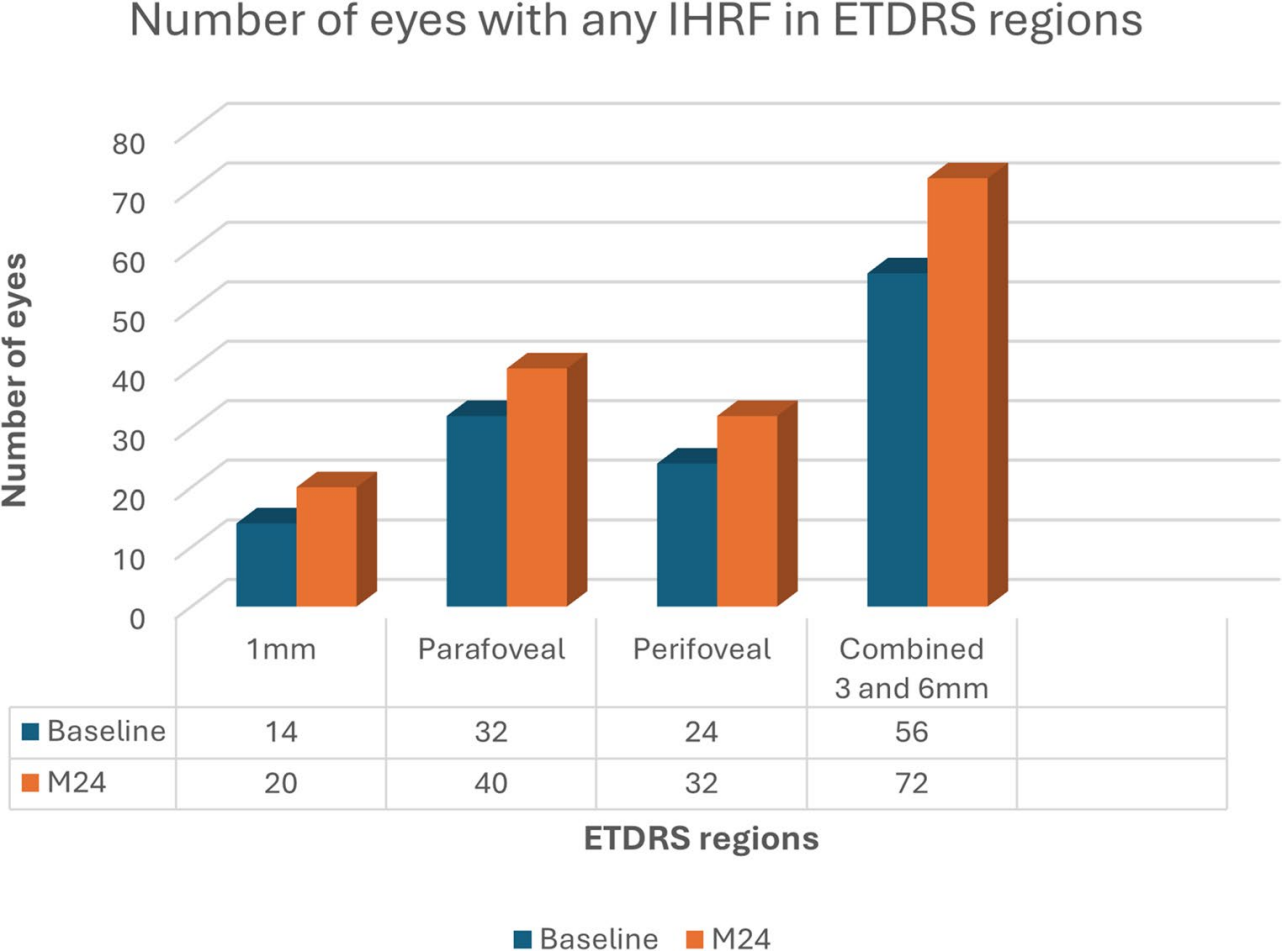
Bar plot illustrating the number of eyes with any IHRF in various ETDRS regions

With regards to the absolute number of IHRF (Table 1), a higher frequency of IHRF was noted in the parafoveal (1–3 mm ring; average of 2.51 IHRF per eye) and perifoveal (3–6 mm ring; average of 1.57 IHRF per eye) region, compared to the CSF (average of 0.83 IHRF per eye). The IHRF count was also significantly higher at M24 compared to baseline in both the parafoveal (average of 6.42 per eye; P *= 0.001*) and perifoveal (average of 3.93 per eye, *P* = 0.02) regions. Furthermore, the number of eyes with any IHRF in the parafoveal (*P* < 0.001), perifoveal (*P* = 0.001), and combined parafoveal and perifoveal rings (*P* < 0.001) were significantly higher when compared to those in the CSF, at both the baseline and M24 (Fig. 3). Two eyes with the IHRF in the CSF at baseline demonstrated an absence of the lesions in the same region at M24.

**Table 1:** Comparison of the transverse distribution of intraretinal hyperreflective foci (IHRF)

**IHRF frequency in ETDRS regions**	**Baseline**	**24 months**	***p***
**Total cohort**			
CSF [sum (range ± SD)]	41 (0–10 ± 1.84)	89 (0–25 ± 4.44)	0.07
Parafoveal [sum (range ± SD)]	123 (0–20 ± 3.76)	315 (0–31 ± 7.88)	**< 0.001**
Perifoveal [sum (range ± SD)]	77 (0–16 ± 3.01)	193 (0–45 ± 8.01)	**0.02**
**Eyes that progressed to Late AMD**			
CSF [sum (range ± SD)]	18 (0–5 ± 1.31)	35 (0–10 ± 2.85)	0.27
Parafoveal [sum (range ± SD)]	79 (0–20 ± 4.78)	193 (0–31 ± 9.03)	**0.01**
Perifoveal [sum (range ± SD)]	23 (0–10 ± 2.1)	112 (0–45 ± 10.22)	**0.045**
**Eyes that did not progress to Late AMD**			
CSF [sum (range ± SD)]	23 (0–10 ± 2.20)	54 (0–25 ± 5.46)	0.15
Parafoveal [sum (range ± SD)]	44 (0–9 ± 2.24)	122 (0–26 ± 6.19)	**0.01**
Perifoveal [sum (range ± SD)]	54 (0–16 ± 3.55)	81 (0–20 ± 5.21)	0.07
**IHRF density in ETDRS regions**	**Baseline**	**24 months**	***p***
Density CSF (range ± SD) in mm^−2^	1.06 (0-12.73 ± 2.34)	2.31 (0-31.84 ± 5.66)	0.17
Density Parafoveal (range ± SD) in mm^−2^	0.39 (0-3.14 ± 0.59)	1.02 (0- 4.93 ± 1.25)	0.85
Density Perifoveal (range ± SD) in mm^−2^	0.07 (0-0.75 ± 0.14)	0.18 (0- 2.12 ± 0.37)	**< 0.001**
**Eyes that progressed to Late AMD**			
Density CSF (range ± SD) in mm^−2^	0.99 (0- 6.36 ± 1.67)	1.93 (0- 12.73 ± 3.63)	0.31
Density Parafoveal (range ± SD) in mm^−2^	0.54 (0- 3.18 ± 0.76)	1.33 (0- 4.93 ± 1.43)	**< 0.001**
Density Perifoveal (range ± SD) in mm^−2^	0.04 (0- 0.47 ± 0.09)	0.22 (0- 2.12 ± 0.48)	**< 0.001**
**Eyes that did not progress to Late AMD**			
Density CSF (range ± SD) in mm^−2^	0.72 (0-6.36 ± 1.67)	2.16 (0-31.84 ± 6.5)	0.32
Density Parafoveal (range ± SD) in mm^−2^	0.22 (0- 1.43 ± 0.32)	0.67 (0- 3.82 ± 0.90)	0.62
Density Perifoveal (range ± SD) in mm^−2^	0.10 (0- 0.75 ± 0.17)	0.15 (0-0.94 ± 0.25)	**< 0.001**

*IHRF *Intra-retinal hyper reflective foci;* ETDRS* Early Treatment Diabetic Retinopathy; *CSF* Central subfield, *1 mm in diameter*,* centered at the fovea; Parafoveal: Region outside the 1 mm circle*,* bound by a circle 3 mm in diameter; Perifoveal: Region outside the 3 mm circle*,* bound by a circle 6 mm in diameter; SD: Standard deviation; AMD: Age-related macular degeneration; Density: Density of IHRF lesions in the corresponding ETDRS regions (counts/mm*^*2*^*).*

With regards to the density of IHRF (Table 1), at baseline, the density was higher in the CSF when compared to the parafoveal region and was lowest in the perifoveal rings. The density was higher at M24 in all the regions, but the increase was statistically significant only in the perifoveal ring (*p* < 0.001). This significant increase of IHRF density in the perifoveal ring was observed in both the subgroup that progressed to late AMD (*p* < 0.001) and the subgroup that did not (*p* < 0.001). For eyes, that progressed to late AMD, however, the increase in IHRF density was also significant in the parafoveal ring (*p* < 0.001).

The average distance of IHRF lesions to the foveal center demonstrated a marginal but nonsignificant numerical increase for the overall cohort (1298.61 μm vs. 1309.74 μm, *P* = 0.88). For eyes that progressed to late AMD, the numerical increase in IHRF distance to the foveal center was of greater magnitude but still did not reach statistical significance (1101.72 μm at baseline vs. 1264.98 μm at M24, *P* = 0.08). In contrast, among eyes that did not progress to late AMD, the proximity of IHRF lesions to the foveal center decreased numerically, though this also did not reach statistical significance. (1472.22 μm vs. 1349.34 μm, *P* = 0.25). When the maximum distance of IHRF rather than the average distance was considered, the distance from the fovea center was noted to increase significantly in the overall cohort (1612.69 μm vs. 1889.71 μm, *P* = 0.009), and in eyes that progressed to late AMD (1456.69 μm vs. 1957.82 μm, *P* = 0.005). The eyes that did not progress showed a numerical increase in distance which did not reach statistical significance (1750.69 μm vs. 1829.46 μm, *P* = 0.54) at M24. (Table 2).

**Table 2:** Distance metrics of IHRF lesions and AMD progression

	**Baseline**	**M24**	*p*
**Total cohort**			
Min distance (range ± SD)	1040.08 (78-2669 ± 739.75)	766.26 (25-1907 ± 519.97)	**0.003**
Max distance (range ± SD)	1612.69 (78-3026 ± 782.98)	1889.71 (728–3604 ± 728.78)	**0.009**
Average distance	1298.61	1309.74	0.88
Median distance	1246.64	1115.5	0.09
**Eyes that progressed to late AMD**			
Min distance (range ± SD)	841.17 (78-2605 ± 617.43)	701.86 (165–1875 ± 500.54)	0.22
Max distance (range ± SD)	1456.69 (78-2714 ± 734.93)	1957.82 (728–3205 ± 740.60)	**0.005**
Average distance	1101.72	1264.98	0.08
Median distance	1044.78	1131.26	0.36
**Eyes that did not progress to late AMD**			
Min distance (range ± SD)	1216.03 (79-2669 ± 792.54)	823.23 (25-1907 ± 530.10)	**0.007**
Max distance (range ± SD)	1750.69 (104–3026 ± 798.12)	1829.46 (856–3604 ± 712.76)	0.54
Average distance	1472.77	1349.34	0.25
Median distance	1425.21	1101.55	**0.005**

*IHRF *Intra-retinal hyper reflective foci*; ETDRS *Early Treatment Diabetic Retinopathy*; all the values are measured in µm*

Despite this difference in distribution over time, logistic regression analysis demonstrated that the distance of IHRF from the foveal center at baseline was not a risk factor for progression to late AMD (Table 3).

**Table 3:** Regression analysis of IHRF distance metrics and the risk of progression to late AMD

Univariate regression analysis					Multivariate regression analysis		
	**OR**	**95% CI**	**p**		**OR**	**95% CI**	**p**
Min distance	0.99	0.99–1.00	0.09		1.002	0.99–1.007	0.35
Max distance	1	0.99–1.00	0.2		1.002	0.99–1.006	0.26
Average distance	0.99	0.99–1.00	0.07		0.99	0.98–1.003	0.24
Median distance	0.99	0.99–1.00	0.07		0.99	0.99–1.004	0.77

## Discussion

In this analysis of eyes with iAMD and IHRF from the Amish Eye Study, we observed that while the absolute number of IHRF were greatest in the parafoveal, the density was highest in the CSF. IHRF did increase throughout the macula over a two-year period, but this increase was only statistically significant in the perifoveal region. However, in eyes that progressed to late AMD, a significant increase in IHRF density was also observed in the parafoveal ring. Notably, IHRF tended to distribute more eccentrically over time in eyes that progressed to late AMD.

This analysis of the transverse distribution provides complementary information to our previous analysis of the axial distribution of IHRF. In our previous study, we observed that IHRF in eyes with AMD preferentially accumulated in the outer retina over time [16], although a few IHRF could be noted in the inner retina. Importantly, in the prior analysis, we observed that IHRF closest to the RPE appeared to be associated with a higher risk for progression to atrophy, whereas IHRF in the inner retina did not appear to increase the risk. Based on this result, we speculated that the cellular origin of inner and outer retinal IHRF might differ, and perhaps outer IHRF corresponded to migrating dissociated RPE cells, whereas inner retinal IHRF might have a microglial origin.

In contrast to the baseline axial distribution of IHRF, the baseline transverse distribution of IHRF did not appear to impact the risk of progression to late AMD over two years. Having said that, it is notable that a greater increase in IHRF density in the parafoveal ring was observed in eyes that progressed to late AMD. In addition, among eyes that progressed to late AMD, the maximum distance of IHRF from the foveal center also significantly increased compared to eyes that did not progress. A possible explanation for this observation is that late AMD tends to develop centrally or para-centrally and with loss of the RPE in these regions, and these atrophic regions may have less propensity for demonstrating IHRF, whereas more peripheral regions without manifest atrophy may be more likely to elaborate IHRF.

A recent study by Saßmannshausen et al. (2023) showed a similar topographic distribution pattern of the IHRF when analyzed in a cross-sectional study [28]. They reported the greatest preponderance of IHRF in a region 0.5–1.5 mm eccentric to the fovea (average 5.2 lesions), followed by the region 1.5–3 mm region (average 2.7 lesions), and > 3 mm from the fovea (average 0.5 lesions), in that order. The group also reported outer nuclear layer (ONL) thinning in the regions overlying IHRF. A similar trend was noted for the IHRF located over the large sub-RPE drusen. A similar pattern relating to the mean IHRF thickness was observed by Waldstein and coworkers in a post-hoc analysis of the contralateral eyes of the patients enrolled in HARBOR trial [8]. Furthermore, Nassisi et al. reported a significant correlation between IHRF area and drusen volume (one of the OCT-based risk factors for AMD progression) in a zone 3 mm eccentric from the foveal center in a cohort of patients with iAMD, both at baseline, and over a follow up period of 1 year [5]. As the study by Saßmannshausen et al. confirmed the ONL thinning directly below IHRF, these reports suggest that the RPE in these regions may be preferentially stressed and prone to elaborate these lesions. The precise mechanism for this preferential localization of IHRF in the macular region remains an area of active investigation, but many lines of evidence point to vascular insufficiency as a contributing factor, as discussed subsequently.

The choriocapillaris (CC) is the primary source of nourishment for the RPE and photoreceptors [29]. CC flow deficits in the macula as measured using swept-source OCT angiography (SSOCTA) have been shown to progressively worse with age, especially beyond 50 years, producing a relatively hypoxic environment [30]. A study by Toto et al. also reported significantly reduced retinal vessel densities in eyes with iAMD, especially in those that exhibited pre-atrophic changes [31, 32]. Similar changes have been reported in both retinal and choroidal circulations in contralateral eyes affected by late AMD [33]. Whether it is the post-receptor neural loss secondary to the vascular insufficiency contributing to the development of AMD or vice-versa, still requires further investigation [33, 34].

A differential choriocapillaris flow pattern with lower flow deficits (FD) in the FCS when compared to the 1–3 mm and 3–6 mm ETDRS rings has been reported in a healthy cohort using SSOCTA [35]. When explored by decade, the FD values in FCS gradually increased after 40 years of age, exceeding the values in the parafoveal 1–3 mm and perifoveal 3–6 mm rings, and remained high thereafter. A similar differential increase in FD with age, especially in the central 1 mm region has been reported by other investigators [36, 37]. Deterioration in the CC flow pattern over time was noted in eyes with apparently stable iAMD, as well as under the lesions like drusen and reticular pseudodrusen [38–40]. Most relevant, our group has also specifically demonstrated more severe CC flow deficits below regions of IHRF and calcified drusen [41, 42]. Furthermore, ischemic choroidopathy has been reported as an important adjunct for the progression from iAMD to late AMD [6]. Choroidal vascularity index (CVI), a ratio between the luminal and the total choroidal area, is known to be a comprehensive biomarker indicating choroidal health [43]. Although various authors have reported inconsistent results when exploring the changes of CVI with age [43–45], a general trend towards increasing CVI with age is generally accepted [35]. Combined together, these various studies highlight the progressive deterioration of the choroidal circulation and choriocapillaris with age and with the development and progression of AMD. The mechanism underlying the observed distribution of IHRF with a predilection for the parafoveal ring, however, is not as clearcut. While the status of CC is relevant, other factors may also need to be considered. Recently, we demonstrated that IHRF are more likely to develop over taller drusen, presumably due to greater separation from the CC which may accentuate the ischemia [46]. The density of overlying photoreceptors may also impact on the severity of RPE ischemia as the photoreceptors are significant consumers of oxygen and nutrients provided by the CC. All of these factors, including others that remain to be defined, may contribute to the observed distribution of IHRF.

Our study is not without limitations, which must be considered when assessing our results. First, a relatively small sample size of patients with iAMD and IHRF and baseline and follow-up limited our power for identifying small changes in IHRF count over time in some regions. Second, although the OCT data was collected in a prospective study, this is a post-hoc analysis and thus is still susceptible to selection bias. Third, the impact of local features such as drusen, subretinal drusenoid deposits, or thick basal laminar deposits were not considered. Fourth, our analysis was based on the Amish population, and while it is a European-based founder population, the findings may not generalize to other populations.

Our study has significant strengths. Despite the concern regarding the generalizability of the Amish, the Amish population has an advantage of being relatively homogenous with regard to both environmental and genetic factors, thus reducing potential confounders. In addition, our analyses were based on data collected using standardized OCT acquisition protocols [47] and analyzed by certified trained graders from the Doheny Image Reading and Research Lab (DIRRL) utilizing a centralized grading protocol.

In summary, we observed a preferential distribution of macular IHRF outside of the foveal central subfield, with the highest density in the CSF. Although the distribution of IHRF at baseline did not additionally impact the risk for progression to late AMD, eyes that progressed to late AMD tended to show a more eccentric distribution of IHRF over time. These observations may provide new insights into the pathophysiology and mechanisms of IHRF development.
